# Attitudes about research among Allied Medical Students enrolled in speech and hearing undergraduate program

**DOI:** 10.12669/pjms.35.3.571

**Published:** 2019

**Authors:** Abdulsalam Alhaidary

**Affiliations:** 1 *Dr. Abdulsalam Alhaidary, PhD, CCC-SLP. Department of Rehabilitation Sciences, College of Applied Medical Sciences, King Saud University, P.O. Box 10219, Riyadh 11433, Saudi Arabia*

**Keywords:** Allied Health Professional, Attitudes, Medical Education, Evidence-based practice (EBP), Research methodology

## Abstract

**Objective:**

To investigate students’ attitudes toward research during their pre-professional training.

**Methods:**

The study collected survey data from 31 undergraduate students enrolled in speech and hearing undergraduate program at the College of Applied Medical Sciences at King Saud University, Riyadh, Saudi Arabia. The data were collected at the beginning of a research methodology course over two consecutive terms. The study used a 32-item questionnaire listed in the Likert scale, and it measured students’ perspectives about research across five sub-scales: usefulness of research, research anxiety, research difficulty, life relevancy of research, and attitude toward research.

**Results:**

The findings showed that students favorably perceived research with average factor ratings ranging from 4.10 to 5.54 out of 7. Also, the study found that the usefulness of research, life relevancy of research, and attitude toward research were correlated highly.

**Conclusions:**

The current study suggests that students’ favorable attitudes toward research may be due, in part, to perceiving research as relevant and useful to their lives and careers.

## INTRODUCTION

Evidence-based practice (EBP) emphasizes providing high quality services to clients based on a process of integrating scientific evidence with clinical experience and client values and preferences.^1^ Clinicians in the field of speech-language pathology and audiology are expected to critically read and evaluate up-to-date clinical literature related to their everyday practice.^2^ Thus, training in research methodology is essential in the education programs of speech-language pathology and audiology to prepare students to be critical consumers of research.^3^ Some researchers have reported that many barriers exist with respect to implementing EBP in the clinical services of speech-language pathology and audiology, such as negative attitudes and the lack of skills to read and integrate research into practice.^4^ Some of these barriers may be related to clinicians’ pre-professional training. According to Irwin, Pannbacker and Lass^5^, “education and training are the foundation for developing and implementing evidence-based practice” (p. 275). Exploring final-year students’ perspectives about research in the field of communication sciences and disorders should, in part, enable a better understanding of these students’ preparedness to use research in their future practice, and also help to create opportunities for improving the curriculum.

Evidence-based practice is based on three factors: scientific research, clinical expertise, and client preferences. According to Straus, Glasziou, Richardson and Hayne,^6^ the process of utilizing clinically relevant research in clinical decisions involves five steps that include

1. Forming a research question based on clinical needs
2. Identifying the best evidence to answer the research question
3. Evaluating the quality of evidence according to the clinical needs
4. Integrating the evidence with client’s values and preferences and with the clinician’s clinical expertise
5. Evaluating the effectiveness and efficiency of the EBP process.

Some speech-language-pathologists (SLPs) and audiologists involve all five steps, whereas others tend to focus on the latter steps as end users.^2^ For all these steps, knowledge and skills in research methodology are essential to effectively implement the EBP process into client care. Particularly, clinicians need to have the skills to identify clinical studies with high quality design.^7^ Many pre-professional curricula in the field of communication sciences and disorders include EBP training and its fundamentals, such as how to research a clinical problem and critically appraise the literature related to clinical questions.^8^

The perception of EBP and research among students enrolled in professional programs in communication sciences and disorders has been investigated by a number of researchers. For example, Spek and associates studied students’ attitudes (self-efficacy) and values about EBP using a questionnaire instrument.^9^ They collected data from SLP first-, second- and third-year students in an undergraduate program in the Netherlands. They found no differences among the three student groups with respect to self-efficacy and values toward EBP. They also found that senior students had more EBP knowledge and skills compared to the other two student groups. However, all three student groups had a low score in self-efficacy toward EBP, i.e., the students’ responses indicated that they did not feel competent about their EBP knowledge and skills. Ratcliff, Swartz and Ivanitskaya had similar findings about senior SLP students having better EBP knowledge and skills.^10^ These researchers studied student information literacy skills (i.e., knowledge about when and where to look for information and how to assess it). They reported better information literacy skills among SLP students working at high academic levels compared to lower academic level students. Cobus-Kuo and Waller examined student information literacy skills and EBP skills by asking students enrolled in an undergraduate speech-language pathology and audiology program to respond to a survey following a 75-minute workshop on information literacy skills that focused on techniques to locate EBP information.^11^ The study found that the participating students had positive attitudes towards information literacy and EBP training, and they found the techniques they learned about to be helpful and useful in their clinical practice. Many students reported that the workshop was their first exposure to EBP training and that training about how to locate and access relevant information should be integrated into their overall training as clinicians. Thus, previous research showed an overall favorable perception toward research, but also highlighted some challenges.

The aim of the present study was to investigate the attitudes towards research of students enrolled in undergraduate programs in communication sciences and disorders. Such an investigation should enable a better understanding of the factors underlying students’ attitudes about research and its significance in their professional lives. Also, the findings of this study should provide some guidance for improving educational programs to enhance students’ research experiences, especially in countries with young and developing speech and hearing professions.

## METHODS

The study sample included 31 undergraduate male students enrolled in a speech and hearing program at the College of Applied Medical Sciences at King Saud University, Riyadh, Saudi Arabia. This undergraduate degree is the entry to the professions of speech-language pathology and audiology in Saudi Arabia. It consists of four years of course work followed by a one year clinical internship. The ages of the students in the present study ranged from 21 to 25 years old. The students’ grade point averages were 2.75 to 3.74 out of 5 for 23 students; 3.75 to 4.49 out of 5 for seven students; and one student did not provide any GPA information. The student data were collected at the beginning of a research methodology course over three consecutive terms using convenience sampling. The study included only students in their final year of the speech and hearing program at King Saud University. The students had no research or minimum research experience, which was limited to distributing a research survey. Only 14 students answered *yes* to the question about their familiarity with the term *evidence-based practice*. Also, eight students indicated that last term they read three to five research articles; 14 reported that they had read one to two research articles; and nine reported that they had not read any research articles last term. Student participation was voluntary, and the participating students were not paid. The study received ethics approval from the Research Ethics Committee of the College of Applied Medical Sciences, King Saud University, Riyadh, Saudi Arabia.

### Instrument

The study questionnaire included two sections. The first section gathered background information about gender, age, parental education, family socioeconomic status, academic information, and previous research experiences. Also, it included two questions about the number of research articles read last term and whether the student was familiar with the term *evidence-based practice* (EBP). The second section included the instrument used in the present study—the attitudes toward research (ATR) scale is a self-report measure of 32-items listed in the Likert scale developed by Papanastasiou to explore students’ attitudes towards research.^12^ The scale is available in an English and Greek version, and the present study used the English version. Students were asked to rate each statement on a scale of 1 to 7 (1 standing for *strongly disagree* and 7 standing for *strongly agree*). The scale measured five factors:

1. Usefulness of research (research usefulness)
2. Research anxiety
3. Research difficulty
4. Life relevancy of research (relevance to life)
5. Attitude towards research (positive research predisposition).^12^

Factor one (usefulness of research) measured how students perceived research as a useful tool for their professional career; it consisted of nine items. Factor two (research anxiety) measured anxiety and feelings of stress towards research; it consisted of eight items. Factor three (research difficulty) searched for problems that students encounter with research; it consisted of three items. Factor four (life relevancy of research) measured how students perceived research as relevant to their everyday living; it consisted of four items. Factor five (attitude toward research) measured students’ feelings and interests about research; it consisted of eight items.^12^

### Data analysis

The students’ attitudes towards research were examined by using a survey research design. Each item had a potential score from 1 to 7; a high score meant a high level of agreement with the statement and vice versa. Thirteen items were negative statements and were recorded so their score represented the same level of agreement like the other 19 items. The score for each factor was calculated by averaging the responses of the subject to the factor items (i.e., providing a factor score). Also, the total score for each subject was calculated by adding the factor scores from all five factors, which could range from 5 to 35 (i.e., providing an overall scale score). The obtained factor scores were treated as ordinal data. Internal consistency was calculated using Cronbach’s alpha coefficient. Considering the qualitative nature of the data, Friedman’s analysis of variance (ANOVA) and Wilcoxon tests were conducted to compare the five factors to each other. Kendall’s tau (τ) also was conducted to examine the correlation among the five factors. IBM SPSS Statistics for Windows (Version 24.0., IBM Corp.) was used to analyze the data.

## RESULTS

**Fig.1:**
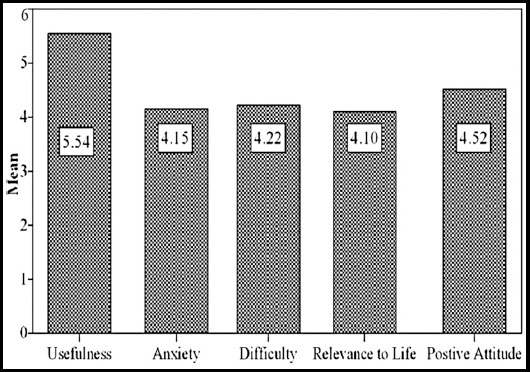
The mean score for each scale factor (factor score).

The ATR scale had an overall reliability coefficient of 0.94, which suggests that the scale items had a high internal consistency. Fig.1 shows the mean score for each factor. Also, Table-I shows the average score for each scale item and the average score for each factor (factor score) The overall scale score calculated by summing the factor scores (i.e., average responses from each factor item) was 22.52, and the range was from 28.67 to 8.93 out of a possible range of 5 to 35. The scale rating of participants significantly differed across the five factors, χ^2^(4) = 38.82, p < 0.0001. Wilcoxon tests were conducted to compare the factors to each other. The factor scores of usefulness of research (Mdn = 5.56) was significantly higher than the factor scores of the other factors: research anxiety (Mdn = 4.38), z = -4.302, p < .0001; research difficulty (Mdn = 4.33), z = -3.774, p < .0001; life relevancy of research (Mdn = 4.25), z = -4.410, p < 0.0001; and attitude toward research (Mdn = 4.63), z = -4.655, p < .0001. Also, usefulness of research was related significantly to life relevancy of research (τ =.327, p = 0.013) and attitude toward research (τ =0.593, p < 0.0001.). Life relevancy of research also was related significantly to attitude toward research (τ =.421, p = 0.001). No other significant findings were found.

**Table-I:** Average Score for Each Scale Item and Average Score for Each Factor (Factor Score).

Factor	Items	Average score	
Usefulness of Research	Research is useful for my career.	6.03	5.54
	Research is connected to my field of study.	5.61	
	Research should be indispensable in my professional training.	5.00	
	Research should be taught to all students.	5.77	
	Research is useful to every professional.	6.23	
	Research is very valuable.	5.65	
	I will employ research approaches in my profession.	5.03	
	The skills I have acquired in research will be helpful to me in the future.	5.81	
	Research is as useful as theory.	4.71	
Research Anxiety	Research makes me nervous.	4.52	4.15
	Research makes me anxious.	4.52	
	Research is stressful.	3.45	
	Research scares me.	5.13	
	Research is a complex subject.	3.77	
	Research is complicated.	3.84	
	Research is difficult.	3.61	
	I feel insecure concerning the analysis of research data.	4.33	
Research Difficulty	I have trouble with statistics.	4.32	4.22
	I find it difficult to understand the concepts of research.	4.55	
	I make many mistakes in research.	3.77	
Life Relevancy of Research	I use research in my daily life.	2.84	4.10
	Research-orientated thinking plays an important role in my daily life.	3.97	
	Research does not apply to my personal life.	4.65	
	Research is irrelevant to my life.	4.94	
Attitude Toward Research	I love research.	3.97	4.52
	I enjoy research.	4.19	
	I like research.	4.16	
	I am interested in research.	4.39	
	Research is pleasant.	4.19	
	Research is interesting.	4.68	
	Most students benefit from research.	5.68	
	I have to study the details of research procedures carefully.	4.87	

## DISCUSSION

The goal of the present study was to examine students’ attitudes towards research, students who were enrolled in an undergraduate program in communication sciences and disorders in Saudi Arabia. The overall impression from the results of the present study is that the participating students favorably perceived research. The average factor ratings ranged from 4.10 to 5.54 out of 7, which suggest a good level of agreement with respect to students’ positive attitudes towards research. This finding is consistent with the results of studies done by other disciplines.^12^-^16^ For example, using an ATR scale, Bell and Clancy surveyed postgraduate students—enrolled in social work in a university in the United Kingdom—about their attitudes toward research learning.^14^ The researchers reported that their participating students had a positive attitude toward research with average scores of and 4.3 for two groups of students. Also, the usefulness of research factor received a higher rating compared to the other factors of the ATR scale. The research is useful to every professional and research is useful for my career items received the highest ratings of 6.23 and 6.03, respectively. These findings suggest that these students viewed research to be a useful tool for their professional career. A similar finding also was reported by Bell and Clancy in which the usefulness of research factor received 5.36 and 5.41 ratings from two groups of students.^14^ Also, the current study found that the usefulness of research factor was related to the life relevancy of research factor and attitude toward research factor; and the life relevancy of research factor was related to the attitude toward research factor. These findings are consistent with Papanastasiou’s results that found a high correlation of usefulness of research with the life relevancy of research and attitude toward research; and a high correlation between the life relevancy of research to a positive attitude toward research.^12^ However, the current study did not find a correlation of the usefulness of research with research anxiety and research difficulty, which Papanastasiou found to be weak—correlations between the usefulness of research and research anxiety (0.363) and correlations between the usefulness of research and research difficulty (0.290).^12^

Overall, the present study found that students had a positive attitude towards research, with correlations among three factors (usefulness of research, life relevancy of research, and attitude toward research), which suggests that students’ positive attitudes towards research may be due to their perception of research as relevant and useful to their lives and careers. Teaching approaches that make learning materials relevant and usefulness may increase student interest toward research. One way to make research methodology course relevant for students is to make learning meaningful. For example, meaningful learning focuses on using teaching techniques that link and integrate previous student knowledge and experiences into new learning materials.^17^ Using similar teaching approaches can promote student learning of research methodology and also increase their research engagement in the future. These teaching approaches can encourage undergraduate students to pursue further research studies toward a doctor of philosophy (PhD), which would help to address the PhD shortage in the field of communication sciences and disorders.^18^-^20^ Additional research with a larger sample is needed to further investigate the relationships among the factors of usefulness of research, life relevancy of research, and attitude toward research in students’ research training.

## CONCLUSION

The current study showed that allied medical students enrolled in speech and hearing undergraduate program have favourable perception of research. The positive attitude toward research may be due to their perception of research as relevant and useful to their lives and careers. Making teaching research methodology course relevant for students can enhance their learning experiences and research interests. An emphasis on knowledge about research methodology continues to grow. It requires clinicians to have the skills not only to participate in research activities but to be able to critically evaluate and integrate scientific information from different sources to make sound clinical decisions.
